# Bacteremia Due to *Clostridium Difficile*: Case Report and Review of the Literature

**DOI:** 10.4137/ccrep.s2204

**Published:** 2009-02-17

**Authors:** Cherag Daruwala, Giancarlo Mercogliano, Gary Newman, Mark J. Ingerman

**Affiliations:** 1 Gastroenterology Fellow, Lankenau Hospital, 252 Lankenau Medical Bldg. East, 100 E Lancaster Ave., Wynnewood, PA 19096.; 2 Gastroenterology Medical Staff, Lankenau Hospital, 252 Lankenau Medical Bldg. East, 100 E Lancaster Ave., Wynnewood, PA 19096.; 3 Infectious Diseases Medical Staff, Lankenau Hospital, 164 Lankenau Medical Bldg. East, 100 E Lancaster Ave., Wynnewood, PA 19096.

**Keywords:** *Clostridium difficile*, Crohn’s disease, bacteremia

## Abstract

**Objective:**

The purpose of this study is to report a case of *C. difficile* bacteremia in a Crohn’s disease patient and to review the literature on previously reported cases.

**Methods:**

Searches of MEDLINE and PubMed databases were made.

**Results:**

We report the first case of *C. difficile* bacteremia in a Crohn’s disease patient. There are 15 other reported cases of *C. difficile* bacteremia reported in the literature. We found that the majority of patients (10 of 15 patients) had polymicrobial bacteremia and that the overall mortality rate is significant, with 6 of 15 reported patients dying.

**Conclusion:**

In conclusion, we find that *C. difficile* bacteremia is associated with a significant mortality rate and it would seem prudent to consider aggressive antibiotic therapy.

## Introduction

*Clostridium difficile* is the primary cause of pseudomembranous colitis and a major cause of antibiotic-associated diarrhea.^1^ In the original report of *C. difficile* published in 1935 the bacterium was named “the difficult clostridium” because early attempts at isolation were unsuccessful and it grew slowly in culture.^3^

*C. difficile* produces an enterotoxin (toxin A) and a cytotoxin (toxin B). Toxin A has been shown to be the cause of diarrhea and pseudomembranous colitis.^4^

*C. difficile* has rarely been reported to cause extraintestinal disease.^1^ The role of toxins A and B in extracolonic manifestations of *C. difficile* remains unclear. We report a case of *C. difficile* bacteremia in a Crohn’s disease patient and review the literature on previously reported cases.

## Methods

A review of the published literature on *C. difficile* bacteremia was done using MEDLINE and PubMed databases. Searches were conducted to find articles from 1966–2008. Medical subject headings used to search the databases included *C. difficile*, including subheadings of bacteremia, extraintestinal disease and Crohn’s disease, as well as a keyword search using “*C. difficile* bacteremia.” Titles and abstracts of potentially relevant articles were reviewed by a single author.

## Case Report

We describe the case of a 50-year-old white male with small bowel Crohn’s disease initially admitted with nausea and abdominal distention secondary to a small bowel obstruction. The patient has a 30 year history of Crohn’s disease involving the jejunum and terminal ileum with multiple proximal small bowel strictures. He has had an appendectomy and back surgery in the past but was never treated surgically for his Crohn’s disease. He was on maintenance therapy with Azulfidine and Azathioprine. The patient was started on Infliximab in November 2005 after multiple admissions for small bowel obstruction. The patient was changed to Adalimumab in May 2007 for patient convenience and difficulty related to obtaining regular intravenous access. He denied any recent antibiotic use.

Computerized tomography scanning on admission demonstrated a small bowel obstruction with thickened and edematous small bowel in the right lower quadrant 8–10 cm from the ileocecal valve with a small amount of ascites and no evidence of abscess. A nasogastric tube was placed for decompression and the patient was placed on solumedrol 20 mg intravenous (iv) every eight hours along with aggressive iv hydration and pain management with hydromorphone. The patient initially improved on hospital day number one. On the morning of hospital day number 2, the patient was reported to be febrile to 39.4 ºC and tachycardic. The patient was complaining of increased pain and on exam had significantly increased tenderness with absent bowel sounds. At that time, the blood culture drawn on admission was reported as growing *Escherichia coli, Enterococcus fecalis and Klebsiella oxytoca.* The patient was started on intravenous antibiotics (ampicillin/sulbactam and gentamicin) and taken for an emergent laparotomy. He was found to have a perforation with a free abdominal abscess and a partial small bowel obstruction of the jejunum. The patient underwent a small bowel resection with jejunojejunal anastamosis and a right hemicolectomy with ileocolonic anastamosis and ileostomy. The pathology revealed a T4N1 poorly differentiated adenocarcinoma of the jejunum. The patient did well clinically post-op however a routine blood culture drawn for fever on post-op day number one grew *Clostridium difficile*. The patient denied significant diarrhea. Subsequent stool studies sent for *Clostridium difficile* toxins A/B were negative. At that time, the patient had received four days of antibiotics. The patient was maintained on pipercillin-tazobactam. All other follow-op blood cultures were unremarkable. The patient received a total of 21 days of intravenous antibiotics (6 days of ampicillin/sulbactam and gentamicin followed by 15 days of pipercillin-tazobactam). The remainder of his post-op course was unremarkable and he made a full recovery.

## Discussion

We report the first case of *C. difficile* bacteremia in a Crohn’s disease patient. There are 15 other reported cases of *C. difficile* bacteremia reported in the literature that are summarized in Table 1. The prevailing theory regarding the pathophysiology of *C. difficile* bacteremia is that the colonic wall inflammation associated with pseudomembranous colitis permits transient bacteremia to develop.

The overall mortality rate is significant, with 6 of 15 reported patients dying. In terms of the demographics, 11 of the 15 patients were male and the age range was from neonate to age 69. A high proportion (4 of the 15 patients) had an underlying malignancy. Unfortunately, *C. difficile* stool toxin was sent in only 7 of the patients. The stool toxin was positive in 5 out of the 7 patients. *C. difficile* Associated Diarrhea (CDAD) was reported in 6 out of the 7 patients. Two out of the 5 patients with positive stool toxin died. The majority of patients (10 of 15 patients) had polymicrobial bacteremia. Four of the reported cases occurred postoperatively.

Recent antibiotic use was found to be a significant risk factor. Antibiotic use leads to an alteration of the intestinal microflora, leading to overgrowth of endogenous *C. difficile* or allowing colonization by nosocomial *C. difficile*. Only 12 of the case reports comment on antibiotic exposure (9 of 12 patients had antibiotic exposure). Cephalosporins were the most common class of antibiotics that these patients were exposed to.

Information on therapy is available on 11 of the patients. The activity of various drugs against *C. difficile* according to the Manual of Clinical Microbiology is summarized in Table 2. Among the cases reviewed, 4 were treated with metronidazole (2 of which died). The specification of oral versus intravenous therapy was incomplete. It is important to note that the two patients who died were both neutropenic leukemic patients. There were 7 patients that were treated (at least in part) with vancomycin and they all survived except one. Two of these patients were treated exclusively with oral vancomycin and they both survived. One of the patients was treated only with intravenous vancomycin and survived. There was also one patient treated with both oral and intravenous vancomycin who survived. The remaining three patients were treated with a regimen that included other antibiotics. In our case, the patient was successfully treated with pipercillin-tazobactam.

*C. difficile* is a ubiquitous organism that can be found in the environment and exposure to the organism is common. It has been estimated that 15%–25% of adults become colonized after admission to the hospital.^16^ There is also growing literature to support a strong link between inflammatory bowel disease and *C. difficile* infection. Previous studies have demonstrated that 5%–20% of patients admitted with an IBD flare will have *C. difficile* infection.^16^ The growing literature support for the link between IBD flares and *C. difficile* infection along with the significant mortality associated with *C. difficile* bacteremia highlight the importance of this topic.

In conclusion, we find that *C. difficile* bacteremia is associated with a significant mortality rate. *C. difficile* Associated Diarrhea (CDAD) was reported in 6 out of the 15 patients. Not surprisingly, the majority of patients had recent antibiotic exposure. We found a high proportion of patients were male. We also found that the majority of patients had a polymicrobial bacteremia. Therefore, it is unclear if *C. difficile* is the primary pathogen. In terms of treatment, it would seem prudent to consider aggressive antibiotic therapy given the high mortality rate.

## Figures and Tables

**Table 1:** Previous Reported Cases of *Clostridium difficile* Bacteremia.

Reference	Sex/Age	Clinical presentation	Stool *C. difficile*	Other organisms isoloated	Antibiotic exposure	Treatment	Outcome
#^5^	M/5	URI	NR	None	NR	NR	NR
#^6^	M/68	Cirrhotic admitted with bacteremia, ascites, and spleenic abscess	NR	None	None	Penicillin G	Died
#^7^	M/neonate	Necrotic small bowel with peritonitis and bacteremia	NR	Staphylococcus epidermedis (likely procedural contaminant)	Ampicillin and kanamycin	Surgery, ampicillin and kanamycin	Died
#^8^	M/65	Toe gangrene with septicemia	Negative	Bacteroides fragilis	Cefuroxime	Oral vancomycin	Survived
#^9^	F/35	AML patient admitted with neurtopenic fever/sepsis	Positive	Bacteroides sp, group D steptococci	Cefotaxime + Gentamicin	Metronidazole (iv) Oral vancomycin	Died
#^9^	F/69	ALL patient admitted with neutropenic fever/sepsis	Positive	Bacteroides sp, Escherichia coli	Metronidazole Ampicillin Cloxacillin Cotrimazole Gentamicin	Metronidazole	Died
#^10^	M/62	Splenic abscess with bacteremia	NR	Pseudomonnas paucimobilis (spleen only)	Pipercillin Netilmycin	Metronidazole Cefoxitin	Survived
#^11^	M/39	Oropharyngeal cancer patient admitted with mandible radionecrosis	Positive	Escherichia coli Enterococcus fecalis Bacteroides vulgatos	None	Metronidazole (iv) Vancomycin(iv) Pefloxacin	Survived
#^1^	F/85	Nosocomial *C. diff.* colitis complicated by bacteremia	Positive	Enterococcus fecalis	Ticacillin/Clavulanic acid	Vancomycin(iv) Gentamcin	Survived
#^12^	M/17	Duchenne musculai dystrophy patient admitted with partial small bowel obstructionr	NR	Candida Parapsilosis	NR	NR	Survived
#^12^	F/33	Cervical cancer patient admitted with pelvic abscess and rectovaginal fistula after radiation	NR	*Clostridium* cadaveris Bacteroides melaninogenicus	NR	NR	Died
#^12^	M/77	Perforated sigmoid diverticulum	NR	Eubacterium lentum	NR	NR	Died
#^13^	M/3	Tonsilitis followed by pericarditis and diarrhea	NR	None	Amoxicillin/Clavulonic acid, Cefixime	Vancomycin (iv) Survived	
#^14^	M/18	Abdominal pain and diarrhea after course of antibiotics for exudative sore throat	Positive	None	Erythromycin	Oral Vancomycin	Survived
#^14^	M/78	Admitted after trauma, treatment for aspiration pneumonia complicated by *C. difficile* bacteremia	Negative	None	Cefuroxime Amikacin	Vancomycin (oral and iv)	Survived

**Notes:** URI, upper respiratory infection; NR, not reported; AML, acute myelogenous leukemia; ALL, acute lymphocytic leukemia; IV, intravenous.

**Table 2:** Activity of various drugs against *C. difficile*.

Antimicrobial agent	CLSI MIC breakpoint (μg/ml)		*C. difficile* % susceptibility

	Susceptible	Intermediate	
Ampicillin	0.5	1	26%
Amoxicillin-clavulanate	4/2	8/4	100%
Pipercillin-tazobactam	32/4	64/4	100%
Ticarcillin	32	64	100%
Clindamycin	2	4	56%
Vancomycin	8	16	100%
Imipenem	4	8	94%
Linezolid	2	4	91%
Metronidazole	8	16	100%
Trimethoprim-sulfamethoxazole	32	64	26%
Trovfloxacin	2	4	86%

Clinical and Laboratory Standards Institute (CLSI) approved method M11-A6(50a); data from Wadsworth Anaerobic Bacteriology. Johnson EA, Summanon P, Finegold SM. *Manual of Clinical Microbiology*, 9th ed. 2007; 904–905.
